# Non-adherence to medication regimens among older African-American adults

**DOI:** 10.1186/s12877-017-0558-5

**Published:** 2017-07-25

**Authors:** Mohsen Bazargan, James Smith, Hamed Yazdanshenas, Masoud Movassaghi, David Martins, Gail Orum

**Affiliations:** 1 Charles R. Drew University of Medicine & Science, 1731 East 120th Street, Los Angeles, CA 90005 USA; 2 University of California, Los Angeles, CA USA; 3 Keck Graduate Institutes, School of Pharmacy, Claremont, CA USA; 4 Department of Family Medicine, Los Angeles, CA USA; 5 Public Health Program, Los Angeles, CA USA

**Keywords:** Non-adherence, Medications, African-Americans, Regimen complexity, Medication knowledge

## Abstract

**Background:**

Despite concerns about racial differences on adherence to prescribed medication rigimens among older adults, current information about nonadherence among underserved elderly African Americans with co-morbidities is limited. This study examines the association between adherence to drug regimens and an array of medication-related factors, including polypharmacy, medication regimen complexity, use of Potentially Inappropriate Medications (PIM), and knowledge about the therapeutic purpose and instructions of medication use.

**Methods:**

Four-hundred African Americans, aged 65 years and older, were recruited from South Los Angeles. Structured, face-to-face interviews and visual inspection of participants’ medications were conducted. From the medication container labels, information including strength of the drug, expiration date, instructions, and special warnings were recorded. The Medication Regimen Complexity Index (MRCI) was measured to quantify multiple features of drug regimen complexity. The Beers Criteria was used to measure the PIM use.

**Results:**

Participants reported taking an average of 5.7 prescription drugs. Over 56% could not identify the purpose of at least one of their medications. Only two-thirds knew dosage regimen of their medications. Thirty-five percent of participants indicated that they purposely had skipped taking at least one of their medications within last three days. Only 8% of participants admitted that they forgot to take their medications. The results of multivariate analysis showed that co-payment for drugs, memory deficits, MRCI, and medication-related knowledge were all associated with adherence to dosage regimen of medications. Participants with a higher level of knowledge about therapeutic purpose and knowledge about dosage regimen of their medications were seven times (CI: 4.2–10.8) more likely to adhere to frequency and dose of medications. Participants with a low complexity index were two times (CI: 1.1–3.9) more likely to adhere to the dosage regimen of their medications, compared with participants with high drug regimen complexity index.

**Conclusions:**

While other studies have documented that non-adherence remains an important issue among older adults, our study shows that for underserved elderly African Americans, these issues are particularly striking. A periodic comprehensive assessment of all medications that they use remains a critical initial step to identify medication related issues. Assessment of their disease and medication related knowledge (e.g., therapeutic purposes, side-effects, special instructions, etc.) and their ability to follow complicated medication regimens and modification of their drug regimens requires inter-professional collaboration.

## Background

Non-adherence to medication is an alarming problem in the United States healthcare system causing an excess cost of over $170 billion annually [1, 2]. Non-adherence to prescription medication can lead to an increase in morbidity, mortality, and healthcare cost [3]. Adherence to prescribed medications is extremely important to ensure the efficacy of medical treatment regimens and more positive health outcomes. Poor medication adherence is relatively common [3–5]. Studies have consistently shown that 20–30% of medication prescriptions are never filled and that, on average, 50% of medications for chronic disease are not taken as prescribed by their provider [6]. Measuring medication adherence is challenging because adherence is an individual patient behavior [7]. Medication non-adherence can occur in different ways, such as not filling the prescription, not taking medication at all, missing doses, taking the wrong amount, taking medication at the wrong time of day, not taking it as prescribed (e.g., with or without food), purposefully discontinuing it for a period of time, or stopping it altogether [4, 8].

Several recent meta-analysis and systematic reviews examined the impact of investigational interventions to improve medication adherence among older adults found a modest main effect of medication adherence and limited improvement in clinical outcomes [9–13]. These systematic reviews and meta-analysis studies unanimously suggest additional empirical research to optimize interventions among elderly populations. Particularly, medication non-adherence among underserved minority populations is not well described in the literature, despite being a major cause of morbidity and mortality among this segment of our population [14, 15]. Indeed, racial differences in adherence to prescribed medication regimens among minority elderly have been previously reported in several studies conducted in US [16–21]. It is suggested that factors that actually change minority patients’ medication-taking practices must be re-examined [15]. Re-examination of correlates of low medication adherence and their interactions among minority underserved population not only guide health service researchers to design more effective interventional strategies, it will also help health providers to better recognize patients at higher risk for chronic disease-related outcomes [22].

Several conceptual models have been used to explain the barriers to medication adherence and drug regimens. Most of these frameworks identified three major components including patient, provider, and health system related factors [13]. Our non-clinic, community-based study focused on medication and patient related factors. We adapted a conceptual model suggested by Gellad and colleagues that categorized barriers to medication adherence among older adults into three major groups, including drug-related, patient-related, and other factors [23]. Gellad and his colleagues documented that major drug-related factors that may impact medication adherence include adverse effects, medication regimen complexity, polypharmacy, and drug interactions [23]. Specifically, medication regimen complexity has been identified as one of the main root causes of patients’ non-adherence [24]. Additionally, those major factors that are patient-related include suboptimal health literacy, lack of disease-related knowledge, and limited knowledge about therapeutic purposes of the medications. Knowledge about the therapeutic purpose of medication is directly related to adherence. A recent study among older adults discharged from hospital shows that only 28% of patients, at the first follow-up, and 25% at the second, understood the reasons for their medications [25].

Older African-American adults, compared to Caucasians, are disproportionally affected by many chronic medical conditions for which multiple prescriptions and treatments are required [26]. Several studies have documented that older African-American adults adhere less than Caucasian patients and suggest that this multiple prescription factor may explain differences in clinical outcomes [17, 27–32]. Although older African-American adults face unique challenges, there is little understanding of medication-related issues among this population, and even less on interventions designed to mitigate their lack of knowledge and adherence to drug regimens. High rate of polypharmacy, medication duplication, and use of Potentially Inappropriate Medication (PIM) among underserved older African American adults all remain major issues [33, 34] that may affect adherence and intended health outcomes among this segment of our population. Studies focusing on medication adherence among older African American adults need to assess the impact of these factors on types of non-adherence behaviors to customize interventions appropriately. In addition, before remedial action can be taken, patients with complex management regimens [24] or other medication-related issues such as medication duplication, PIM use, and medication interactions must be identified.

This study examines the association between adherence to drug regimens and a spectrum of medication-related factors, including polypharmacy, medication regimen complexity, use of PIM, and knowledge about their therapeutic purpose and instructions of proper medication use. We employed rigorous methods to measure the complexity of drug regimens, adherence to drug regimens, and knowledge regarding therapeutic purpose of each prescribed medications that they use.

## Methods

The data was collected in 2013–14 from 400 community-dwelling, underserved, older adult, self-identified African Americans from 16 predominantly African-American churches located in Service Planning Area 6 (SPA6) of Los Angeles County. SPA6 is home to over one million residents and disproportionately impacted by health disparities compared to the rest of Los Angeles County [35]. In addition to posting flyers announcing the proposed project at respective churches, the coordinator of this project assisted church leaders to convene pre- or post-Sunday sermon meetings to introduce the project to the parishioners. Two co-authors of this study, both trained physicians, conducted the face-to-face interviews in a private room at participating sites. This study was approved by the Charles R. Drew University of Medicine and Science Institutional Review Board. Less than 5% of individuals who were approached refused to participate. Written informed consent was obtained from participants.

### Measurement

We conducted a comprehensive brown bag medication assessment to capture several medication-related factors including polypharmacy, medication regimen complexity, use of potentially inappropriate medications, and medication duplications. The validity and superiority of medication-related information obtained through the brown bag method have been documented. Indeed, when the focus of medication examination is on assessing participants’ medications at a specific point in time, brown bag data may provide more complete information than pharmacy records [36].

#### Comprehensive medication review

Medication use was determined through visual inspection of containers by trained interviewers. The respondents were asked to show interviewers all medications taken within the 2-week period prior to interview. Label information or, in the absence of a drug name on the label, a description of the physical appearance of each drug was recorded. In addition, the interviewer transcribed from the container label all other information including medication names and its generic equivalents, strength of the drug, expiration date, instructions, special warnings, providers’ information, etc. Furthermore, additional questions were asked about the use and method of procurement of individual drugs as each drug was displayed to the respondent. All of these medications have been classified according to the 2013 Prescription Drug List of United Healthcare & Affiliated Companies. Any duplication of medications was documented at the time of the survey interview and was later evaluated by the research team pharmacist.

#### Adherence to drug regimen (outcome variable)

To examine adherence to drug regimens, participants were given opportunities to examine each medication and its container while answering seven questions. Four out of seven items were adopted and modified from Morisky Medication Adherence Scale (MMAS) [37, 38]. These four items document participants’ self-report about “forget”, “skip”, “reduce”, or “stop taking” prescribed medications within the last 3 days prior to the interviews for ***each medication separately***. In reviewing the development and implementations of Morisky scale and its modification, Tan and colleagues reported that there is still some space for additional improvement of the face validity and content validity of the MMAS [39]. They indicated that MMAS is not fully able to comprehensively assess the reasons or predictors of medication adherence. In addition, they indicated that patients with multiple comorbidities may hold diverse medication beliefs and medication taking behaviors for different diseases and medications. However, MMAS is designed for patients to solely focus on one specific disease state at one time [39]. The other three items of our adherence survey instrument measure participants’ self-report adherence to special instructions for taking each medication (food, drink, and physical activities).

### Medication-related variables

#### Medication regimen complexity index (MRCI)

This study employed the MRCI developed and validated by George and colleagues, a tool for quantifying multiple features of drug regimen complexity [40]. Complexity of medication regimen is a theoretical concept independent of clinical, pharmacologic, and demographic factors. The MRCI quantifies complexity of regimens according to the dosage forms, dosing frequencies, and additional directions. The MRCI has three sections with each representing a different facet of complexity. The MRCI has been designed as an open index since there is no upper limit for the number of drugs that could be prescribed for a patient or the number of additional instructions possible in a particular regimen. The MRCI is a reliable and valid tool with potential applications in both clinical practice and research [40]. All components of the MRCI have been independently found to influence patient adherence [24].

#### Potentially inappropriate medication (PIM)

By visual inspection and transcription of all medication container labels, our team pharmacists used the 2012 American Geriatrics Society revised Beers Criteria [41] to document the number of PIM use for each participant. All of the inappropriately prescribed and over-the-counter medications were divided in two categories: 1) “Use Conditionally,” and 2) “Avoid.”

#### Polypharmacy

Polypharmacy has been frequently mentioned as one of the major medication-related issues among older adults with co-morbidities [42]. Polypharmacy is characterized as the use of multiple medications for the treatment of a single, or several, coexisting diseases [33]. Indeed, the term polypharmacy has been used with different definitions including but not limited to unnecessary drug use, medication use without indication, and use of multiple medications [42]. We defined polypharmacy merely based on the number of medications used by participants [43]. Number of medications used was categorized into two groups: less 1–4, and ≥5 medications.

#### Medication duplication

Medication duplication was assessed by the drug inventory method. All medications that were taken by participants within two weeks prior to the interviews were classified according to the 2013 Prescription Drug List of United Healthcare & Affiliated Companies.

### Patient-related variables

#### Demographic characteristics

Self-reported demographic information included age, sex, marital status defined as partnered (married or living with a partner) or un-partnered (divorced, widowed, separated or never married), and education.

#### Smoking status and alcohol use

Smoking status and alcohol use during the past year were self-reported. Smoking status was categorized into three groups: current, former, and never smoked. In addition, alcohol consumption was categorized based on number of drinks per day (none, 1–2, and ≥3 drink per day).

#### Memory deficit

Self-appraisal of memory capability was measured using the Meta-Memory Questionnaire-Ability (MMQ-Ability) instrument. The MMQ-Ability contains 20 everyday memory situations, such as remembering appointments, names, and telephone numbers. For each item, respondents indicated the frequency with which each mistake occurred on a 5-point scale (all the time = 0, often, sometimes, rarely, never = 4) with higher scores indicating better subjective memory ability (α = 0.908). This instrument has excellent validity and reliability among healthy older adults and have been recommended for both clinical and research settings [44].

#### Co-morbidity

Participants self-reported “Yes” or “No” to a list of medical conditions, such as arthritis, back pain, kidney disease, stroke, hypertension, etc.

#### Knowledge of therapeutic purpose of prescribed medications

This element was assessed by asking following questions:

1. What are the reasons for which this medication was prescribed for you?
2. How much of this medication did your doctor say you should take?
3. How often did your doctor say you should take this drug?
4. Do you need to take or avoid any types of food or drink while taking this medication?
5. Do you need to avoid any activities while taking this medication?
6. Do you know if there would be any side effects from this medication?

The number of drugs that a participant did not attempt to identify was recorded and a nonidentification score computed. Also, for the drugs that subjects did attempt to identify, their perceived purpose of each prescription drug was evaluated based on a comparison by a registered pharmacist with all therapeutic indications used in clinical practice and subsequent computation of a misidentification score. To create an index or indices to be used for the multivariate analysis, a factor analysis with Varimax rotation was performed using the above mentioned six items. Factor analysis identified two meaningful dimensions. First factor includes items that measure how often and why (therapeutic knowledge) medication should be used and the second factor was associated with items that assess the participants’ knowledge about side effects and whether any activity, food, or drink must be avoided or taken with medications.

### Other independent variables

#### Required co-payment

Participants were asked if any co-payment is required to fill a prescription.

#### Number of providers

Number and description of pharmacists and providers used by participants were recorded from the label of medication containers.

#### Statistical analysis

Descriptive statistics were used to measure the frequency, mean, and standard deviation of all variables. Furthermore, we used the chi-square test to examine correlates of non-adherence to durg regimens. In addition, multiple logistic regression was employed to examine the correlates of adherence to medication adjusting for demographic characteristics and other relevant factors. We selected two independent variables: 1) adherence to dosage regimen (dose and interval) of prescription medications and 2) skipping or taking decreased medication dosages or forgetting to take a medication. We excluded from the multivariate logistics analysis several variables that did not show a significant association with outcome variables at the bivariate level to avoid a harmful multicollinearity. Secondly, we reported two models for each outcome to minimize multicollinearity because we detected a strong association between drug regimens and the polypharmacy variable. Models one and three include the drug regimens complexity index and excludes the polypharmacy. Models two and four exclude the complexity index and include only polypharmacy index. We utilized a *p*-value <0.05 to identify statistically significant differences. To avoid multicollinearity, a diagnostic test was performed in multivariate analysis to examine inter-correlation among independent variables.

## Results

### Characteristics of sample

This sample included 400 African-American individuals who were between the ages of 65 to 94 years (Mean 73.5 ± 7). More than 21% (84) were 80 years of age or older. One out of four participants reported having no high school diploma. Almost 65% of participants were women. Only 20% (72) of the sample were currently married or lived with a companion. Our data shows that the number of reported chronic illnesses by participants ranged from zero to 17, with the average at just over five (5.2 ± 3.01). Nineteen percent (76) of participants reported at least eight chronic conditions. Over 85% (341) reported that they have been diagnosed with hypertension. Almost 37% (147) of participants were diagnosed with diabetes mellitus.

Participants were taking an average of 5.7 (range: 0 to 18; SD: 3.02) prescription drugs. More than 40% of participants were taking zero to four medications, whereas, 49% and 11% of participants were taking five to nine and at least 10 prescription medications, respectively. The mean value of the medication regimen complexity index (MRCI) was 15.1 (SD = 8.2; Minimum = 2.5 and Maximum = 55.5). Thirty-eight percent (153) of participants received prescriptions from three or more providers. Furthermore, the medication containers showed that 28% (113) of participants used at least two pharmacies to fill their prescriptions. Using the 2012 American Geriatrics Society revised Beers Criteria, [41] our data showed that 70% (278) of participants engaged in PIM use and used at least one medication that was classified as “Avoid” (27%) and “Use Conditionally” (43%).

### Non-adherence to medications

**Fig. 1:**
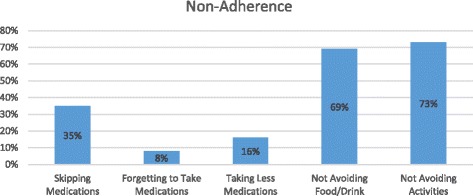
Patterns of Nonadherence to Drug Regimens

Figure 1 reports the patterns of non-adherence to drug regimens among our sample. Thirty-five percent (138) of participants indicated that they purposely had skipped taking at least one of their medications within last 3 days (Fig. 1). Interestingly, only 8% (32) of participants admitted that they forgot to take at least one of their medications within the last 3 days prior to the interviews. While only 3% (11) of participants indicated that they took more medications than were prescribed by their providers, 16% (63) reported that they were intentionally taking fewer medications than they were instructed. Furthermore, our data show that a surprisingly low rate of adherence to instructions regarding food/drink and activities while taking medications. More than 69% (275) and 73% (290) of participants were not adhering to the instructions for specific food/drink and activities as indicated on their medication container, respectively. When frequency and dose of medications reported to be taken by participants were compared with the instructions provided by the medication containers, findings indicate that 47% (188) of our participants are non-adherent with at least one of their medications, within the last 3 days. Almost 30% (119), 10% (39) and 7.5% (30) were not taking one, two, or three or more of their medications according to the container instructions, respectively.

### Knowledge of medications

**Fig. 2:**
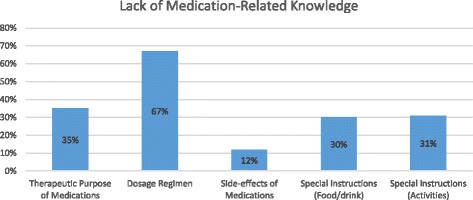
Medication-Related Knowledge

Almost 65% (258) of participants could not identify the purpose of at least one of their prescription medications that they had taken within 2 weeks prior to the interview. Almost two-thirds (263) of the participants knew the appropriate dosage of medications that were prescribed for them. Strikingly, only 12% (48) of participants could state the major side-effects of their prescribed medications. In addition, only 29% (116) and 30% (120) of participants knew whether they should avoid any types of “food or drink” and “activities” while taking their medications, respectively (Fig. 2).

### Bivariate correlates of adherence to drug regimens

Table 1 reports bivariate correlates of adherence to drug regimens with all other variables. Eight out of 16 variables were significantly associated with the adherence to frequency and dose of medications used (column 3). This table reports that the co-payments for medications, number of providers, number of co-morbidities, memory deficits, medication-related knowledge, complexity index, and polypharmacy all were associated with adherence to dosage regimen of medications. In addition, bivariate correlates of skipping medication intentionally are also reported in Table 1 (column 4). Female participants with higher number of providers and co-morbidities, those with medication duplications, those who were identified for using medications that Beers criteria indicated should be avoided, those who using at least five medications (polypharmacy) and those with complex medication regimens were more likely to intentionally avoid taking their medications. Table 1 (columns 5–8) report other bivariate correlates of adherence to additional items of adherence to drug regimens.

**Table 1:** Characteristic of Study Sample by adherence to Drug Regimens (*N* = 400)

Characteristic of Sample	N (%)	Non-Adherence to at least One Medication Regimens N (%)					
		Dose and interval	Deciding to skip	Forgot to take	Taking Less	Failed to follow food/drink instructions	Failed to follow activity instructions
Gender							
Male	141 (35)	70 (50)	**37 (26)**	12 (9)	23 (14)	101 (72)	96 (69)
Female	259 (65)	118 (46)	**101 (39)**	20 (8)	40 (15)	189 (73)	179 (69)
Age							
65–74	241 (61)	115 (48)	88 (37)	20 (8)	44 (18)	201 (83)	163 (67)
≥ 75	157 (39)	73 (46)	50 (32)	12 (8)	19 (12)	139 (89)	112 (71)
Education							
No high school diploma	99 (25)	50 (49)	31 (32)	7 (7)	14 (4)	77 (79)	**76 (78)**
High school diploma	301 (75)	138 (46)	107 (36)	25 (8)	49 16)	213 (71)	**199 (66)**
Marital Status							
Not married	322 (80)	146 (46)	32 (41)	6 (7)	11 (14)	57 (73)	51 (65)
Married/living with Partner	78 (20)	42 (54)	106 (33)	26 (8)	52 (16)	233 (73)	224 (70)
Co-payment for RX							
No	83 (21)	**49 (59)**	28 (34)	7 (8)	10 (12)	52 (63)	**49 (59)**
Yes	317 (79)	**139 (44**)	110 (35)	25 (8)	53 (17)	238 (76)	**226 (72)**
Number of Providers							
1–2	247 (62)	**96 (39)**	**71 (29)**	18 (7)	32 (13)	**161 (66)**	**159 (65)**
≥ 3	153 (38)	**92 (60)**	**71 (46)**	14 (9)	31 (20)	**129 (84)**	**116 (76)**
Number of Pharmacists							
1	287 (72)	128 (45)	96 (34)	19 (7)	40 (14)	202 (71)	188 (66)
≥ 2	113 (28)	60 (53)	42 (37)	13 (12)	23 (20)	88 (78)	87 (77)
Number of Co-morbidity							
≤ 3	128 (32)	**49 (39)**	**25 (20)**	7 (6)	10 (8)	85 (67)	76 (60)
4–7	195 (49)	**90 (46)**	**78 (40)**	17 (9)	40 (21)	143 (74)	141 (73)
≥ 8	77 (19)	**49 (64)**	**35 (45)**	8 (10)	13 (17)	62 (81)	58 (75)
Smoking Status							
Current	64 (16)	31 (48)	22 (34)	5 (8)	13 (20)	**36 (56)**	40 (63)
Never	334 (84)	157 (47)	116 (35)	27 (8)	50 (15)	**254 (76)**	237 (71)
Alcohol Use (Drinks/day)							
No/Occasionally	355 (89)	164 (46)	119 (34)	27 (8)	54 (15)	257 (72)	246 (69)
Yes	43 (11)	24 (56)	19 (44)	5 (12)	9 (21)	33 (77)	29 (67)
Memory Deficit							
Not Noticeable	176 (45)	**69 (39)**	53 (30)	11 (6)	23 (13)	124 (70)	130 (74)
Mild to Moderate	160 (40)	**79 (49)**	64 (40)	15 (9)	31 (19)	119 (74)	102 (64)
Severe	61 (15)	**39 (63)**	21 (34)	6 (10)	9 (15)	46 (75)	42 (69)
Medication Knowledge							
*A. How many/often*							
Correct	260 (65)	**66 (25)**	89 (34)	36 (14)	24 (9)	181 (70)	177 (68)
Incorrect	140 (35)	**122 (88)**	49 (36)	27 (20)	8 (6)	109 (79)	98 (71)
*B. Purpose of Rx*							
Correct	140 (35)	**50 (36)**	42 (30)	20 (14)	13 (9)	**95 (68)**	**83 (59)**
Incorrect	258 (65)	**136 (53)**	96 (37)	43 (17)	19 (7)	**195 (76)**	**192 (74)**
Complexity Index							
< 10	108 (27)	**30 (28)**	**20 (19)**	7 (6)	**10 (9)**	**84 (78)**	**57 (53)**
≥ 10	290 (73)	**158 (54)**	**118 (41)**	25 (9)	**53 (18)**	**256 (88)**	**218 (75)**
Polypharmacy							
0–4	161 (40)	**56 (34)**	**42 (26)**	25 (15)	12 (8)	**100 (62)**	**99 (61)**
≥ 5	237 (60)	**134 (57)**	**98 (41)**	40 (17)	22 (9)	**190 (80)**	**176 (74)**
Medication Duplication							
No	185 (46)	81 (44)	**53 (29)**	13 (7)	20 (11)	128 (70)	119 (65)
Yes	215 (54)	107 (50)	**85 (40)**	19 (9)	43 (20)	162 (77)	156 (73)
PIM (AVOID)							
None	292 (73)	132 (46)	**92 (32)**	24 (8)	41 (14)	**204 (70)**	196 (68)
At Least One	108 (27)	56 (52)	**46 (43)**	8 (7)	22 (20)	**86 (80)**	79 (73)

Note: Bolded values are statistically significant (*P* ≤ .05)

### Multivariate correlates of medication use

Table 2 reports the results of multiple logistic regression adjusted odd ratios (OR) between independent variables and the adherence to medication regimens. Adjusting for demographic characteristics and other related variables, model one and two show that 1) co-payment for the medications, 2) memory deficits, 3) medication-related knowledge indices were all associated with adherence to dosage regimen of prescribed medications. In addition, drug regimen complexity index was associated with the outcome variable, however, polypharmacy did not show statistically a significant association with the adherence to medications Participants demonstrating a high complexity index were two times (OR = 2.02; CI: 1.10–3.90) more likely to non-adhere to the dosage regimen of their medications, compared with participants with low drug regimen complexity index. These two models show that knowledge about medication instructions and therapeutic purpose of medications contributed to dosage regimen adherence of prescribed medications to a far greater extent than other factors.

**Table 2:** Multivariate Logistic Analysis of Correlates of Non-Adherence to Drug Regimens (*N* = 400)

Independent variables	Non-Adherence with dosage regimen OR (95% CI) Model 1	Non-Adherence with dosage regimen OR (95% CI) Model 2	Taking less - skip or forget OR (95% CI) Model 3	Taking less - skip or forget OR (95% CI) Model 4
Gender				
Female	1	1	1	1
Male	1.16 (0.68–1.98)	1.21 (0.71–2.07)	1.59 (0.99–2.55)	1.53 (0.95–2.46)
Age				
≥ 75	1	1	1	1
65–74	0.69 (0.41–1.16)	0.71 (0.43–1.19)	0.79 (0.51–1.23)	0.78 (0.50–1.21)
Education				
No high school diploma	1	1	1	1
High school diploma	0.72 (0.39–1.32)	0.73 (0.40–1.41)	0.85 (0.51–1.43)	0.92 (0.55–1.54)
Co-payment for RX				
No	**1**	**1**	1	1
Yes	**2.94 (1.59–5.26)** ^**a**^	**2.86 (1.54–5.26)** ^**a**^	1.19 (0.69–2.04)	1.11 (0.65–1.89)
Memory Deficit				
Not Noticeable	**1**	**1**	1	1
Mild to Moderate	1.27 (0.60–2.63)	1.27 (0.60–2.70)	0.87 (0.36–1.32)	0.68 (0.36–1.32)
Severe	**2.5 (1.18–5.26)** ^**b**^	**2.63 (1.25–5.56)** ^**a**^	0.88 (0.46–1.69)	0.88 (0.46–1.69)
Medication Knowledge				
Factor 1 (Dosage/Purpose)	**6.76 (4.24–10.8)** ^**a**^	**7.35 (4.53–11.9)** ^**a**^	0.93 (0.74–1.16)	0.93 (0.73–1.17)
Factor 2 (Side Effects/Other Instructions)	1.33 (0.99–1.78)	**1.49 (1.09–2.05)** ^**b**^	**1.28 (1.02–1.62)** ^**c**^	**1.29 (1.03–1.64)** ^**c**^
Medication Duplication				
No	1	1	1	1
Yes	1.25 (0.71–2.17)	0.97 (0.58–1.64)	1.08 (0.66–1.72)	0.87 (0.56–1.37)
PIM				
None	1	1	1	1
At Least One	1.23 (0.70–2.32)	1.19 (0.67–2.08)	0.76 (0.47–1.23)	0.76 (0.92–1.25)
Medication Regimen Complexity Index				
< 10	**1**	NA	**1**	NA
≥ 10	**2.02 (1.10–3.90)** ^**c**^	-	**2.43 (1.31–4.50)** ^**b**^	-
Polypharmacy				
0–4	NA	1	NA	1
≥ 5	-	1.09 (0.60–1.97)	-	1.66 (0.99–2.80)
-2 Log Likelihood	391.2	395.5	491.9	491.6
Nagelkerke R-Square	0.438	0.428	0.11	0.10
% of correctly predicted outcome	77%	76%	66%	66%

Notes: ^a^ indicates *P*-value <001; ^b^ indicates *P*-value < .01; ^c^ indicates *P*-value < .05

RX = Prescription medication

PIM = Potentially Inappropriate medication

Participants with a higher level of knowledge about therapeutic purpose and knowledge about dosage regimen of their medications were almost seven times (OR = 6.76: CI: 4.24–10.8) more likely to adhere to frequency and dose of medications. The odds of being in the group of survey respondents who adhere to frequency and dose of their medications was almost three times (OR = 2.94; CI: 1.59–5.26) higher among individuals who indicated no co-payment for their Rx compared to their counterparts who had a copayment of Rx medications. Finally, as it was expected, participants with a higher level of memory deficit were 2.5 times (OR = 2.5; CI: 1.18–5.26) less likely to adhere to frequency and dose of their prescribed medications.

## Discussion

Examination of the National Health and Nutrition Examination Survey shows that during the last decade, the median number of prescription medications used among older adults doubled from 2 to 4, and that those taking more than 5 medications tripled from 13% to 39% [45]. This increase in medication use was driven, in part, by a higher use of cardio-protective medications used to treat hypertension and hyperlipidemia [45]. The use of these medications has led to substantial improvements in management of common chronic conditions. Despite these successes, suboptimal use of these medications, especially among older African-American adults, prevents their maximal health benefits [46]. Poor adherence to prescribed medications precludes older African American adult patients from the potential benefits of prescription medications and may in fact contribute to the disproportionate burden of morbidity and mortality in this population. Many system-level and individual patient- and prescription-level barriers to medication adherence among the aged have been identified in the literature [47, 48] including but not limited to socio-demographic characteristics, medical comorbidities, cognitive dysfunction, medication side-effects, regimen complexity and polypharmacy. Our study was specifically designed to delineate medication-related modifiable and non-modifiable barriers to medication adherence and to identify barriers most amenable to intervention for improving adherence to prescribed medications among this vulnerable elderly population. While many of the patient barriers to medication adherence identified in this study have been previously described in the literature, relative contribution of medication-related factors of poor adherence to prescription medication among older African-American adults has not been fully elucidated.

Polypharmacy has been frequently mentioned as one of the major medication-related issues among older adults with co-morbidities [42]. Reviewing literature, Pasina and colleagues report that polypharmacy is very common among older adults and is significantly contributes to poor adherence to therapies [25]. Yet, we found no significant independent association between polypharmacy and medication adherence, once other related variables are held constant. However, we found more complex medication regimens were associated with fewer adherences in our sample of older African American Adults. A recent systematic review of studies that focused on clinical outcomes and medication regimen complexity in older adults reports that research into whether medication regimen complexity is associated with nonadherence and hospitalization has produced inconsistent results [49]. This systematic review indicates that moderate-quality evidence from a few studies suggests that medication regimen complexity is associated with nonadherence and higher rates of hospitalization. A Swedish study confirmed that both polypharmacy and drug regimen complexity are associated with unplanned hospitalization among older adults [50]. With no doubt, medication regimen complexity is high in older adults with co-morbidity and additional work is needed to address polypharmacy and to determine how medication regimen complexity influences adherence and clinical outcomes among older adults [51].

Another important medication-related issue that impacts health outcome of older adults is the use of potentially inappropriate medications [41]. A recent systematic review of literature shows that PIM use was associated with a 1.6-fold increased mortality in older adults [52]. A retrospective cohort study using nationally representative data from 2006 to 2010 Medical Expenditure Panel Survey (MEPS) reports that of community-dwelling older adults with prescription medications, 42.6%, had at least one medication filled that met the broad definition of PIM [53]. Our data shows substantially higher use of PIM among this sample of underserved older African American adults. Almost 70% of our sample engaged in PIM use and used at least one medication that was classified as “Avoid” (27%) and “Use Conditionally” (43%) through Beers Criteria. This is substantially higher than previously reported PIM use among a national sample of non-institutionalized older adults. However, multivariate analysis of our data shows no association between medication adherence and PIM that may reflect the lack of knowledge on the purpose and “signetur” (sig:) of the prescription medication.

A final medication and patient related issue that needs special attention is knowledge about medications that patients used. We documented that participants with a higher level of knowledge about the therapeutic purpose of dosage regimen were almost seven times more likely to adhere to their medication. This finding highlights the relative importance of medication knowledge compared to the other non-financial barriers. Other studies documented that patients’ knowledge about the therapeutic purpose of each medication that they used contributes to greater patient incentive following treatment recommendations [25]. In addition, association between health literacy, knowledge of prescribed medications, and adherence to medication among minority population has been documented by other researchers. A recent study has documented that medication adherence in African Americans improves with increasing health literacy [54]. Managing medication adherence by improving the knowledge of elderly adults about their medication has been identified as an effective intervention to improve their adherence to medication regimens. [55]. After all, medication knowledge may be more amenable to intervention to improve medication adherence, especially among older African-American adults.

Our studies did not extensively examine the system and provider-related factors. However, one of the important factors that was included in our study which requires further elaboration is the role of prescription co-payment on adherence. The association of prescription co-payment with poor adherence in this study is consistent with the existing body of literature and may account in part for poorer health outcomes among the older African-American adults [56]. Reviewing 160 articles and abstracts published from January 1974 to May 2008, Eaddy and colleagues documented that 85% of studies showed that an increasing patient share of medication costs was significantly associated with a decrease in adherence [56]. National data shows that implementation of Medicare Part D and the affordable Care Act has improved the rates of cost-related prescription non-adherence among older adults [57]. However, efforts to improve medication adherence for vulnerable populations may benefit from interventions at the level of local pharmacies, as well as medication benefit redesign [58]. It is important to note that cost re-education strategies will not totally eliminate non-adherence. Comparing medication adherence among 149,893 elderly Medicare beneficiaries and 21,204 disabled non-elderly beneficiaries, Zhang and colleague documented that non-adherence to heart failure drugs remains problematic, especially among minority beneficiaries. Even among beneficiaries with close-to-full drug coverage, racial differences remain, suggesting that cost reduction strategies and policies cannot eliminate racial differences [59].

It is imperative to mention several limitations of this study. First, this study used a cross-sectional study design which allowed us to collect data at a single point in time. Therefore, it is difficult to draw causal inferences. As a simple example, we documented that participants with a higher level of knowledge about the therapeutic purpose of prescription dosage regimen were almost seven times more likely to adhere to their medication. However, without a longitudinal data and causal identification strategy, it is challenging to prove that this association is a causal relationship. Secondly, older African-American adults who participated in this study were selected from local churches that may be different than non-church attenders and may introduce a selection bias. Third, the assumption is made that participants brought in all of the medication vials that they used within 2 weeks prior to the interviews. Lack of access to participants’ medical or pharmacy records limits our ability to validate this assumption. However, several attempts were made prior to the face-to-face interviews to make sure that participants will follow the instructions and bring all their medications with them.

## Conclusion

While other studies have documented that medication adherence remains an important issue among older adults, our study shows that for the underserved elderly African-American community, these issues are particularly striking. We also documented that several medication-related issues among this segment of our population are alarming. Logically, the first step in improving adherence among underserved older adults with multiple comorbidities is to conduct a comprehensive assessment of all their prescribed medications and any other medications that they take. The goal should be to identify issues such as excessive use of medications, drug interactions, inappropriate medications, the use of unauthorized and expired medications, and assessment of the medication regimen complexity. The next step must focus on enhancing health literacy and disease-related knowledge, explaining the therapeutic purposes of medications, side-effects, special instructions, etc. Inter-professional collaboration using a patient-centered approach that promotes appropriate communication between older adult patients and providers is the key for achieving this goal [60–62].
